# Comparison of 5-year outcomes between trabeculectomy combined with phacoemulsification and trabeculectomy followed by phacoemulsification: a retrospective cohort study

**DOI:** 10.1186/s12886-021-01949-9

**Published:** 2021-04-24

**Authors:** Shogo Arimura, Kentaro Iwasaki, Yusuke Orii, Yoshihiro Takamura, Masaru Inatani

**Affiliations:** Department of Ophthalmology, Faculty of Medical Science, University of Fukui, Yoshida, 23-3 Simoaizuki, Matsuoka, Fukui, Japan

**Keywords:** Trabeculectomy, Phacoemulsification, Long-term outcome

## Abstract

**Background:**

We aimed to compare the outcomes of trabeculectomy combined with phacoemulsification and those of trabeculectomy followed by phacoemulsification.

**Methods:**

A total of 141 patients with primary open-angle glaucoma, exfoliation glaucoma, and glaucoma secondary to uveitis glaucoma who underwent trabeculectomy followed by (*n* = 48) or combined with (*n* = 93) phacoemulsification were included. We analyzed data collected from the Collaborative Bleb-Related Infection Incidence and Treatment Study, a prospective cohort study conducted in 34 clinical centers that included 1249 eyes. The main outcome was the cumulative probability of success based on intraocular pressure (IOP) within 5 years. Surgical failure was defined as a case in which additional glaucoma surgery is required or one of the following criteria are met: preoperative IOP > 21 (A), > 18 (B), or > 15 mmHg (C). The secondary outcomes were cumulative probability of success, risk factors of surgical failure, and Δ visual acuity. However, the data on phacoemulsification during the 5-year follow-up were censored.

**Results:**

No significant difference was found in the cumulative probability of success as the main outcome. When the data on phacoemulsification during the 5-year follow-up were censored, the probabilities of success of trabeculectomy followed by phacoemulsification were significantly higher for criteria A (*p* = 0.02), B (*p* <  0.01), and C (*p* <  0.01). Lower preoperative IOP, younger age, and trabeculectomy combined with phacoemulsification were associated with poorer outcome. Trabeculectomy followed by phacoemulsification had significantly worse Δ logMAR visual acuity at 6 and 12 months (*p* <  0.01).

**Conclusion:**

The cumulative probability of success after trabeculectomy combined with or followed by phacoemulsification remained unchanged. Combining phacoemulsification with trabeculectomy adversely affected the cumulative probability of success after trabeculectomy. The visual acuity improvements observed in the early postoperative period after combining phacoemulsification with trabeculectomy disappeared within 5 years.

## Background

Trabeculectomy has been the standard filtering surgery for patients with glaucoma due to uncontrollable intraocular pressure (IOP) [1]. When trabeculectomy is performed in patients with glaucoma, the best timing to perform cataract surgery is difficult to determine because the cataract frequently progresses after trabeculectomy [2–4] and because phacoemulsification is known to adversely affect IOP control after trabeculectomy [5–7]. Recently, we also reported that phacoemulsification performed in patients with a history of trabeculectomy led to poorer surgical outcomes of trabeculectomy alone [8], and trabeculectomy combined with phacoemulsification has poorer surgical outcomes than trabeculectomy alone [9, 10]. In addition, the numbers of patients with glaucoma and cataract are increasing with the aging of society and population growth worldwide [11]. This situation has caused increasing concern about the best timing to perform cataract surgery for patients with a history of trabeculectomy. However, no studies conducted in large cohorts and over long periods have assessed surgical outcomes between trabeculectomy followed by phacoemulsification and trabeculectomy combined with trabeculectomy.

In the present study, we analyzed data collected from the Collaborative Bleb-Related Infection Incidence and Treatment Study (CBIITS), a prospective cohort study conducted in 34 clinical centers that included 1249 eyes [12]. The CBIITS investigated the incidence, severity, and prognosis of bleb-related infections after trabeculectomy with mitomycin C treatment for 5 years. Therefore, the present study aimed to compare long-term outcomes between trabeculectomy followed by phacoemulsification and trabeculectomy combined with phacoemulsification and to identify the risk factors of surgical failure. By clarifying the outcomes, we aimed to help determine whether cataract surgery should be performed at the same time as trabeculectomy or later in clinical settings.

## Methods

### Patient selection

The CBIITS is a multicenter prospective cohort study that aimed to investigate the incidence and risk factors of bleb-related infection. The investigators from the Japan Glaucoma Society voluntarily participated in the CBIITS, in which 34 clinical centers were enrolled as participants. Institutional review board approval from each clinical center was obtained, and written informed consent was obtained from all the participants. The protocol conformed to the tenets of the Declaration of Helsinki. The postoperative ophthalmologic examinations were conducted every 6 months up to 5 years. The CBIITS enrolled 1249 eyes of 1249 patients for 2 years, and the enrollment was completed on March 31, 2007. The study finally included 1098 eyes treated with trabeculectomy.

The institutional review board of Fukui University Hospital, Fukui, Japan, approved the present study, and 141 eyes of 141 patients with primary open-angle glaucoma, exfoliation glaucoma, and glaucoma secondary to uveitis who had participated in the CBIITS were included. We notified and disclosed research information on the homepage to guarantee the opportunity for research participants to refuse. The enrolled patients were divided into two groups, the “trabeculectomy followed by phacoemulsification” group and the “trabeculectomy combined with phacoemulsification” group. “Trabeculectomy followed by phacoemulsification” referred to the performance of lens extraction with phacoemulsification during the 5-year follow-up period after trabeculectomy. In the CBIITS, the local investigators at each center decided the indications for surgery, selection of operative procedure, operative technique, and application of postoperative medication or additional glaucoma treatment, either medically or surgically. However, trabeculectomy was usually performed with 0.04% mitomycin and a fornix- or limbus-based conjunctival incision. Phacoemulsification was performed with a clear corneal or superior conjunctival incision.

In the present study, we included Japanese patients with primary open-angle glaucoma, exfoliation glaucoma, and glaucoma secondary to uveitis (with the anterior chamber angle assessed using slit-lamp biomicroscopy and gonioscopy with an angle mirror, and the fundus evaluated using ophthalmoscopy or fundus photography); who were at least 20 years of age; who underwent trabeculectomy with 0.04% mitomycin C; who had phakic eyes; and who completed the 5-year follow-up without dropouts. We excluded eyes with histories of glaucoma and vitreoretinal surgeries, rupture during phacoemulsification, and any postoperative intraocular surgeries except for glaucoma reoperation or phacoemulsification.

### Main outcome measure

The primary outcome measure was the cumulative probability of success in the Kaplan–Meier survival curve analysis based on IOP. IOP was measured with Goldmann applanation tonometry every 6 months at each center during the 5-year follow-up in the CBIITS. In the present study, surgical failure was defined, before performing the data analysis, on the basis of the following IOP levels with or without antiglaucoma medications: < 20% reduction in preoperative IOP or IOP > 21 mmHg (criterion A), IOP > 18 mmHg (criterion B), or IOP > 15 mmHg (criterion C) [13]. In addition, surgical failure was declared in cases that required reoperation for glaucoma or had loss of light perception or low IOP (≤5 mmHg). Reoperation for glaucoma was comprised of bleb needling > 6 months after trabeculectomy, bleb revision, or additional glaucoma surgery. Laser suture lysis or bleb needling within 6 months after trabeculectomy was not considered as surgical failure because it was a part of the postoperative management for trabeculectomy.

### Secondary outcome measures

The secondary outcomes included three factors. The first factor was the cumulative probability of success in the Kaplan–Meier survival curve analysis, with the same criteria as those of the primary outcome, but the data on phacoemulsification during the 5-year follow-up were censored. The second factor was the risk factors of failure in the first secondary outcome. The third factor was Δ visual acuity (the postoperative visual acuity minus the preoperative visual acuity) during the 5-year follow-up. Visual acuity aggregated in the form of decimal visual acuity with the Landolt chart was converted to a logarithm of the minimum angle of resolution (logMAR) visual acuity in the statistical analysis.

### Sample size

In a previous study [11], the cumulative probability of success after trabeculectomy followed by phacoemulsification appeared to be superior to that of trabeculectomy combined with phacoemulsification when the data on phacoemulsification during the 5-year follow-up were censored. Therefore, we assumed proportional hazards and designed the sample size by using the method of Lakatos et al. [14] to test the difference between the survival functions of the two groups. If the difference in surgical failure rate between the two groups was ≥25% with a two-sided significance level of 0.05 and a power of 0.8, the estimated sample size of at least 105 eyes was considered as essential to detect a significant difference between the two groups.

### Statistical analyses

SPSS version 26.0 (IBM Institute, Inc. Chicago, IL, USA) was used for the statistical analysis. Univariate analyses were performed using the Wilcoxon non-parametrical and chi-square tests for baseline characteristics. The Wilcoxon non-parametrical test with Bonferroni correction was used to determine the Δ visual acuity between the two groups. The 5-year cumulative probability of surgical failure was analyzed using the Kaplan–Meier survival curves and log-rank test. The Cox proportional hazards regression model was used to determine the risk factors of surgical failure. *P* values were considered statistically significant if less than 0.05.

## Results

### Baseline characteristics

Table 1 shows the baseline characteristics. A total of 48 eyes were treated with trabeculectomy followed by phacoemulsification, whereas 93 eyes were treated with trabeculectomy combined with phacoemulsification. Significant differences were observed in the number of eyes with secondary glaucoma due to uveitis (*p* = 0.02), preoperative logMAR visual acuity (*p* <  0.01), and the base of the conjunctival flap (*p* = 0.03) between the two groups. Preoperative IOP (mean ± SD) was not significantly different between the “trabeculectomy followed by phacoemulsification” group (24.0 ± 10.1 mmHg) and “trabeculectomy combined with phacoemulsification” group (24.4 ± 8.0 mmHg; *p* = 0.35).

**Table 1:** Baseline characteristics

	TLE followed by Phaco	TLE combined with Phaco	*p* Value
n	48	93	
Age (years)	64.5 ± 12.0	67.7 ± 9.95	0.197
Sex, male (%)	56.2	53.8	0.86
POAG, n (%)	31 (65)	50 (54)	0.29
EXG, n (%)	8 (17)	7 (8)	0.17
SG, n (%)	9 (19)	36 (39)	0.02^†^
LogMAR VA	0.14 ± 0.28	0.48 ± 0.50	< 0.01^*^
Base, fornix (%)	34.7	54.8	0.03^†^
IOP (mmHg)	24.0 ± 10.1	24.4 ± 8.0	0.35
Glaucoma medication (n)	2.3 ± 1.1	2.7 ± 1.0	0.14

*TLE* Trabeculectomy, *Phaco* Phacoemulsification, *POAG* Primary open-angle glaucoma, *EXG* Exfoliation glaucoma, *SG* Secondary glaucoma, *Posner* Posner-Schlossman syndrome, *LogMAR* Logarithm of minimum angle of resolution, *VA* Visual acuity, *IOP* Intraocular pressure.

^†^Chi-square test

*Wilcoxon non-parametrical test

### Primary outcome

**Fig. 1:**
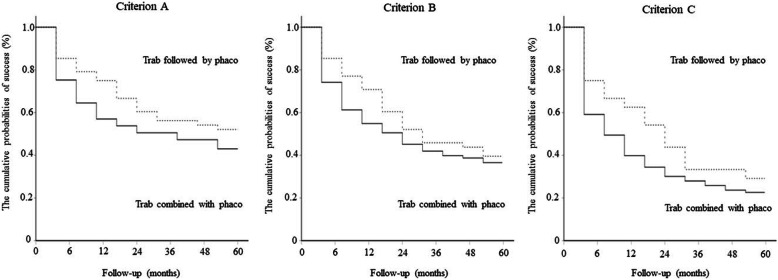
Comparison of the Kaplan–Meier survival curve analysis results between the “trabeculectomy followed by the phacoemulsification” and “trabeculectomy combined with phacoemulsification” groups for criteria **a**, **b**, and **c**. No significant differences were found between the two groups in all the criteria. The data were compared using a log-rank test. Trab = trabeculectomy; Phaco = phacoemulsification

The results of the Kaplan–Meier survival curve analysis between the “trabeculectomy followed by phacoemulsification” and “trabeculectomy combined with phacoemulsification” groups for criteria A, B, and C are shown in Fig. 1. No significant differences were observed between the two groups in any criteria. For criterion A, the cumulative probabilities of success of trabeculectomy followed by phacoemulsification and trabeculectomy combined with phacoemulsification at 5 years were 52.1 and 43.0% (*p* = 0.22), respectively. For criterion B, the 5-year cumulative probabilities of success of trabeculectomy followed by phacoemulsification and trabeculectomy combined with phacoemulsification were 39.6 and 36.6% (*p* = 0.44), respectively. For criterion C, the 5-year cumulative probabilities of success of trabeculectomy followed by phacoemulsification and trabeculectomy combined with phacoemulsification were 29.2 and 22.6% (*p* = 0.14), respectively. Failure due to insufficient IOP reduction was observed in 18 eyes (38%) based on criterion A, 25 eyes (52%) based on criterion B, and 27 eyas (56%) based on criterion C in the “trabeculectomy followed by phacoemulsification” group, and insufficient IOP reduction was observed in 43 eyes (46%) based on criterion A, 49 eyes (53%) based on criterion B, and 64 eyes (69%) based on criterion C in the trabeculectomy alone group (Table 2).

**Table 2:** Insufficient IOP reduction for surgical failure

Criterion	TLE followed by Phaco	TLE combined with Phaco	*p* Value
	*n* = 48	*n* = 93	
A	*n* = 18 (38%)	*n* = 43 (46%)	0.32
B	*n* = 25 (52%)	*n* = 49 (53%)	0.95
C	*n* = 27 (56%)	*n* = 64 (69%)	0.14

The data were analyzed with a chi-square test.

*TLE* Trabeculectomy, *Phaco* Phacoemulsification, *IOP* Intraocular pressure

### Secondary outcomes

**Fig. 2:**
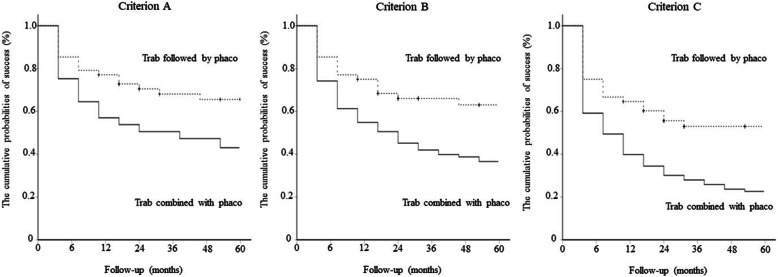
Comparison of the Kaplan–Meier survival curve analysis results between the “trabeculectomy followed by phacoemulsification” and “trabeculectomy combined with phacoemulsification” group for criteria **a**, **b**, and **c**, when the data on phacoemulsification performed during the 5-year follow-up were censored. The probabilities of success for criteria **a**, **b**, and **c** in the “trabeculectomy followed by phacoemulsification” and “trabeculectomy combined with phacoemulsification” groups were 66.7 and 43.0% for criterion A (*p* = 0.02), 64.6 and 37.6% for criterion B (*p* < 0.01), and 54.2 and 22.6% for criterion C (*p* < 0.01), respectively. The data were compared using a log-rank test. Trab = trabeculectomy; Phaco = phacoemulsification

Figure 2 shows that the “trabeculectomy combined with phacoemulsification” group had significantly lower cumulative probabilities of success for criteria A, B, and C (*p* = 0.02, *p* <  0.01, and *p* < 0.01, respectively) than the “trabeculectomy followed by phacoemulsification” group when the data on phacoemulsification during the 5-year follow-up were censored. For criterion A, the 5-year cumulative probabilities of success of trabeculectomy followed by phacoemulsification and trabeculectomy combined with phacoemulsification were 66.7 and 43.0% (*p* = 0.02), respectively. For criterion B, the 5-year cumulative probabilities of success of trabeculectomy followed by phacoemulsification and trabeculectomy combined with phacoemulsification were 64.6 and 37.6% (*p* < 0.01), respectively. For criterion C, the 5-year cumulative probabilities of success of trabeculectomy followed by phacoemulsification and trabeculectomy combined with phacoemulsification were 54.2 and 22.6% (*p* < 0.01), respectively.

### Risk factors of surgical failure

The results of the Cox proportional hazards model when data on phacoemulsification during the 5-year follow-up were censored are listed in Table 3. Trabeculectomy combined with phacoemulsification was consistently associated with poorer surgical outcomes for criteria A (*p* = 0.01), B (*p* = 0.01), and C (*p* < 0.01). Preoperative lower IOP was associated with poorer surgical outcomes for criteria A (*p* < 0.01) and B (*p* = 0.04). Younger age was associated with poorer surgical outcomes for criteria A (*p* = 0.02) and B (*p* = 0.04).

**Table 3:** Hazard ratio analyzed using the multivariate Cox proportional hazards regression models

Factor	Criteria					
	A		B		C	
	HR (95% Cl)	*p* Value	HR (95% Cl)	*p* Value	HR (95% Cl)	*p* Value
Age (years)	0.97 (0.95–0.99)	0.02^*^	0.98 (0.95–0.99)	0.04^*^	0.98 (0.96–1.00)	0.07
Sex, F/M	1,24 (0.75–2.07)	0.41	1.01 (0.62–1.64)	0.96	0.98 (0.64–1.50)	0.93
Preoperative IOP per mmHg	0.94 (0.91–0.98)	< 0.01^*^	0.96 (0.93–0.99)	0.04^*^	1.00 (0.96–1.03)	0.71
Conjunctival incision (Fornix/limbus based)	0.93 (0.55–1.55)	0.77	0.96 (0.58–1.58)	0.86	1.06 (0.69–1.64)	0.80
EXG/POAG	0.58 (0.26–1.29)	0.18	0.67 (0.31–1.47)	0.65	1.07 (0.50–2.31)	0.86
SG/POAG	0.71 (0.28–1.77)	0.46	0.79 (0.32–1.95)	0.61	0.99 (0.42–2.32)	0.98
TLE combined with phaco	2.31 (1.22–4.38)	0.01^*^	2.26 (1.22–4.18)	0.01^*^	2.23 (1.30–3.82)	< 0.01^*^
Number of glaucoma medications	1.04 (0.80–1.35)	0.77	1.06 (0.82–1.35)	0.68	1.12 (0.90–1.40)	0.31

*HR* Hazard ratio, *F* Female, *M* Male, *IOP* Intraocular pressure, *POAG* Primary, *EXG* Exfoliation glaucoma, *SG* Secondary glaucoma due to uveitis, *Posner* Posner-Schlossman syndrome, *TLE* Trabeculectomy, *Phaco p*hacoemulsification.

*Log-rank test

### Δ visual acuity

**Fig. 3:**
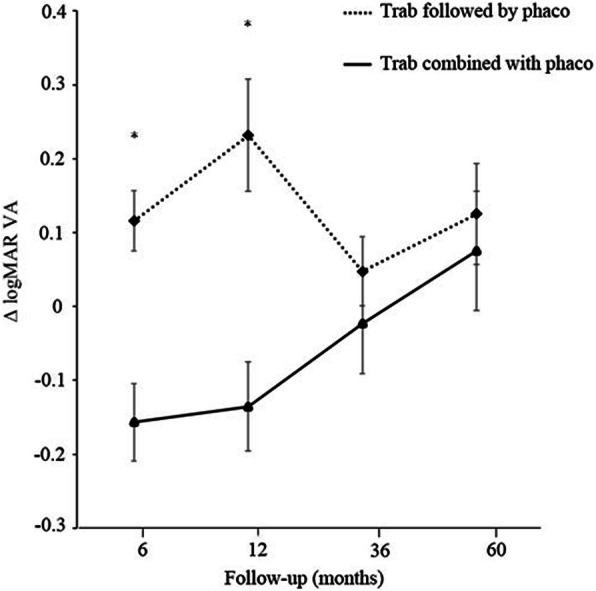
Comparison of Δ logMAR visual acuity between the “trabeculectomy followed by phacoemulsification” and “trabeculectomy combined with phacoemulsification” groups. Trabeculectomy followed by phacoemulsification had higher Δ logMAR acuity at 6 and 12 months (6 months, *p* < 0.01; 12 months, *p* < 0.01). The data (mean ± standard deviation) were compared using the Wilcoxon non-parametrical test with Bonferroni correction. Trab = trabeculectomy; Phaco = phacoemulsification; LogMAR = logarithm of minimum angle of resolution; VA = visual acuity; Δ = postoperative logMAR VA minus preoperative logMAR VA

The 5-year Δ visual acuity of the “trabeculectomy followed by phacoemulsification” and “trabeculectomy combined with phacoemulsification” groups are shown in Fig. 3. The Δ visual acuity in the “trabeculectomy followed by phacoemulsification” and “trabeculectomy combined with phacoemulsification” groups were 0.13 ± 0.25 and − 0.16 ± 0.47 at 6 months (*p* < 0.01), 0.20 ± 0.46 and − 0.15 ± 0.53 at 12 months (*p* < 0.01), 0.06 ± 0.29 and − 0.03 ± 0.60 at 36 months (*p* = 0.12), and 0.11 ± 0.41 and 0.07 ± 0.76 at 60 months (*p* = 0.40), respectively.

## Discussion

In the present study, we found no significant differences in the cumulative probabilities of success between the “trabeculectomy followed by phacoemulsification” and “trabeculectomy combined with phacoemulsification” groups. By contrast, when the data on phacoemulsification performed during the 5-year follow-up were censored, significant differences were observed for criteria A, B, and C between the two groups. The potential risk factors in the Cox proportional hazards model revealed that trabeculectomy combined with phacoemulsification is an independent contributor to surgical failure (criterion A: RR = 2.31, *p* = 0.01; criterion B, RR = 2.26, *p* = 0.01; and criterion C, RR = 2.23, *p* < 0.01; Table 3).

No prospective studies have been conducted in a large cohort with a long follow-up period to investigate the outcome of trabeculectomy combined with phacoemulsification. Ogata et al. prospectively reported that trabeculectomy combined with phacoemulsification (*n* = 25) resulted in inadequate IOP reduction as compared with trabeculectomy alone (*n* = 25) at 1 year [10]. Sacchi et al. retrospectively demonstrated that trabeculectomy alone (*n* = 40) achieved a higher success rate and lower mean IOP at follow-up than trabeculectomy combined with phacoemulsification (*n* = 27) for the patients with a mean follow-up period of 25.7 ± 14.4 months [15]. In another retrospective study, trabeculectomy combined with phacoemulsification had a lower cumulative probability of success than trabeculectomy alone for 4 years among older patients with open-angle glaucoma [11]. In a retrospective study, Donoso et al. showed that the surgical survival curve of trabeculectomy combined with phacoemulsification with intraoperative administration of 5-fluorouracil had no significant difference with that of trabeculectomy followed by phacoemulsification with intraoperative administration of 5-fluorouracil [16]. The results of the present study were consistent with those of previous studies that showed that trabeculectomy combined with phacoemulsification had no significant difference in cumulative probability of success based on IOP as compared with trabeculectomy followed by phacoemulsification or had lower cumulative probabilities of success than trabeculectomy alone. In contrast to trabeculectomy alone, combined phacoemulsification increases the risk of blood–aqueous barrier collapse. The flare value after phacoemulsification returns to normal only after 6 months, but the flare value after trabeculectomy returns to normal only after 4 weeks [17]. In addition, various fibrogenic cytokines such as monocyte chemotactic protein-1 and interleukin-8 are secreted after phacoemulsification [18, 19]. The prolonged inflammation eventually leads to bleb scarring [20].

Although significantly better outcomes in terms of Δ logMAR visual acuity were exhibited in the “trabeculectomy combined with phacoemulsification” group as compared with the “trabeculectomy followed by phacoemulsification” group at 6 and 12 months after surgery, the difference in Δ logMAR visual acuity disappeared 12 months after surgery. In the “trabeculectomy combined phacoemulsification” group, visual acuity improved within a short-term follow-up period after surgery owing to the performance of cataract extraction. Similarly, Lockhead et al. [11] reported a mean gain in the number of lines in the Snellen visual acuity chart from baseline after trabeculectomy combined with phacoemulsification in patients with open-angle glaucoma with at least 1-year follow-up as compared with those who underwent trabeculectomy alone. Choy also retrospectively showed that patients who underwent phacotrabeculectomy had better visual acuity improvement for 3 months after surgery than those who underwent trabeculectomy alone [21]. Park et al. [6] also demonstrated that the mean visual acuity beyond the first postoperative month was significantly better than that at baseline after trabeculectomy combined with phacoemulsification. Similar to phacoemulsification alone, trabeculectomy combined with phacoemulsification also improved visual acuity [22, 23]. Moreover, trabeculectomy alone for phakic eyes triggered the occurrence of lens opacification after surgery [2–4, 24]. The deterioration of visual acuity within 12 months after surgery in the “trabeculectomy followed by phacoemulsification” group seemed to be due to the progression of cataract. Thirty-three of the 48 eyes in the “trabeculectomy followed by phacoemulsification” group underwent phacoemulsification within 36 months. Therefore, the mean Δ logMAR visual acuity was restored at 36 months after trabeculectomy. The difference in lens opacity between the two groups possibly led to the significant outcomes of Δ logMAR visual acuity in the “trabeculectomy combined with phacoemulsification” group within 12 months. The Δ logMAR visual acuity at 60 months was almost equal between the two groups because of the gradual deterioration of visual acuity in the “trabeculectomy combined with phacoemulsification” group. As shown in Fig. 2, the surgical outcomes of the “trabeculectomy combined with phacoemulsification” group were significantly worse than those of the “trabeculectomy followed by phacoemulsification” group when data on phacoemulsification during the 5-year follow-up were censored. Compared with those in trabeculectomy alone, the uncontrolled postoperative IOPs in combined phacoemulsification might have resulted in the gradual deterioration of visual acuity over 5 years.

In the multivariate analysis using a Cox proportional hazards model, two additional risk factors of surgical failure were identified, namely younger age and lower preoperative IOP. Younger age has been considered a risk factor of trabeculectomy failure in many previous studies [25–28]. Vigorous healing response frequently occurs in individuals with a younger age, possibly owing to their thicker Tenon’s capsule [29]. A > 20% IOP reduction in eyes with lower preoperative IOP is difficult to achieve without hypotony [8, 30].

This study had some limitations. First, as this was a cohort study, the participants were not randomly divided into two groups. In fact, the use frequency of fornix-based conjunctival flap was significantly higher in the “trabeculectomy combined with phacoemulsification” group. However, the Cox proportional hazards model showed that the surgical outcomes were not dependent on the base of the conjunctival flap. The incidence of secondary glaucoma due to uveitis was significantly higher in the “trabeculectomy combined with phacoemulsification” group. Eyes with uveitis were associated with cataract [31, 32]. The high incidence of cataract in the uveitic glaucomatous eyes might have caused the selection bias. Second, phacoemulsification, trabeculectomy, and trabeculectomy combined with phacoemulsification were not standardized. The technique used to perform these procedures depends on the discretion of each center. Hence, further prospective randomized studies should be conducted to resolve these limitations.

## Conclusion

In conclusion, the present study showed no significant difference in cumulative probability of success between trabeculectomy followed by phacoemulsification and trabeculectomy combined with phacoemulsification for patients with primary open-angle glaucoma, exfoliation glaucoma, and glaucoma secondary to uveitis glaucoma. However, trabeculectomy combined with phacoemulsification appeared to have a lower cumulative probability of success than trabeculectomy alone. When the data on phacoemulsification during the 5-year follow-up were censored, younger age, lower preoperative IOP, and trabeculectomy combined with phacoemulsification were risk factors of surgical failure. The Δ logMAR visual acuity in trabeculectomy followed by phacoemulsification was significantly higher in the early postoperative period than in trabeculectomy combined with phacoemulsification. However, the Δ logMAR visual acuity had no significant differences between the two groups regardless of the timing of phacoemulsification.

## Data Availability

The datasets used and/or analyzed during the present study are available from the corresponding author on reasonable request.

## Abbreviations

CBIITS: Collaborative Bleb-related Infection Incidence and Treatment Study
IOP: Intraocular pressure
LogMAR: Logarithm of minimum angle of resolution
